# A novel diphtheria toxin‐based bivalent human EGF fusion toxin for treatment of head and neck squamous cell carcinoma

**DOI:** 10.1002/1878-0261.12919

**Published:** 2021-02-20

**Authors:** Zeng Qi, Yue Qiu, Zhaohui Wang, Huiping Zhang, Ling Lu, Yanqiu Liu, David Mathes, Elizabeth A. Pomfret, Dexiang Gao, Shi‐Long Lu, Zhirui Wang

**Affiliations:** ^1^ Division of Plastic and Reconstructive Surgery Department of Surgery School of Medicine University of Colorado Anschutz Medical Campus Aurora CO USA; ^2^ Division of Transplant Surgery Department of Surgery School of Medicine University of Colorado Anschutz Medical Campus Aurora CO USA; ^3^ Department of Otolaryngology School of Medicine University of Colorado Anschutz Medical Campus Aurora CO USA; ^4^ Department of Biostatics School of Medicine University of Colorado Anschutz Medical Campus Aurora CO USA

**Keywords:** diphtheria toxin, EGF, EGFR, fusion toxin, head and neck cancer, HNSCC

## Abstract

Epidermal growth factor receptor (EGFR) is often overexpressed in head and neck squamous cell carcinoma (HNSCC) and represents a top candidate for targeted HNSCC therapy. However, the clinical effectiveness of current Food and Drug Administration (FDA)‐approved drugs targeting EGFR is moderate, and the overall survival rate for HNSCC patients remains low. Therefore, more effective treatments are urgently needed. In this study, we generated a novel diphtheria toxin‐based bivalent human epidermal growth factor fusion toxin (bi‐EGF‐IT) to treat EGFR‐expressing HNSCC. Bi‐EGF‐IT was tested for *in vitro* binding affinity, cytotoxicity, and specificity using 14 human EGFR‐expressing HNSCC cell lines and three human EGFR‐negative cancer cell lines. Bi‐EGF‐IT had increased binding affinity for EGFR‐expressing HNSCC compared with the monovalent version (mono‐EGF‐IT), and both versions specifically depleted EGFR‐positive HNSCC, but not EGFR‐negative cell lines, *in vitro*. Bi‐EGF‐IT exhibited a comparable potency to that of the FDA‐approved EGFR inhibitor, erlotinib, for inhibiting HNSCC tumor growth *in vivo* using both subcutaneous and orthotopic HNSCC xenograft mouse models. When tested in an experimental metastasis model, survival was significantly longer in the bi‐EGF‐IT treatment group than the erlotinib treatment group, with a significantly reduced number of metastases compared with mono‐EGF‐IT. In addition, *in vivo* off‐target toxicities were significantly reduced in the bi‐EGF‐IT treatment group compared with the mono‐EGF‐IT group. These results demonstrate that bi‐EGF‐IT is more effective and markedly less toxic at inhibiting primary HNSCC tumor growth and metastasis than mono‐EGF‐IT and erlotinib. Thus, the novel bi‐EGF‐IT is a promising drug candidate for further development.

## Graphical abstract

In this study, we have developed a diphtheria toxin‐based bivalent human EGF fusion toxin for the treatment of HNSCC with significantly improved efficacy and markedly less *in vivo* off‐target toxicity compared with the monovalent human EGF fusion toxin. The bivalent human EGF fusion toxin is a promising novel drug candidate for the treatment of EGFR‐positive HNSCC.

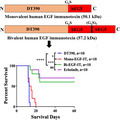

**Abbreviations**
ATP: adenosine triphosphate
bi‐EGF‐IT: bivalent epidermal growth factor fusion toxin
DT: diphtheria toxin
EGF: epidermal growth factor
EGFR: epidermal growth factor receptor
FDA: Food and Drug Administration
G_4_S: four glycine residues and one serine residue
hEGF: human epidermal growth factor
His: histidine
HNSCC: head and neck squamous cell carcinoma
HPV: human papilloma virus
IC_50_: half‐maximal inhibitory concentration
IP: intraperitoneal injection
IT: fusion toxin
IV: intravenous injection
*K*_D_: equilibrium dissociation constant
kDa: kilodalton
m: molar concentration
mAb: monoclonal antibody
MFI: mean fluorescence intensity
mono: monovalent
mono‐EGF‐IT: monovalent epidermal growth factor fusion toxin
nm: nanomolar concentration
*NSG*: NOD/SCID IL‐2 receptor γ^−^^/–^
PE: phycoerythrin
R/M: recurrent/metastatic
SA: streptavidin
scFv: single‐chain variable fragment

## 1 Introduction

Over 90% of head and neck cancers are head and neck squamous cell carcinoma (HNSCC), including cancers derived from the oral cavity, nasopharynx, oropharynx, hypopharynx, and larynx [1]. The annual incidence of HNSCC is ~ 65 000 in the United States and 470 000 worldwide [2, 3]. Smoking, drinking, and the human papillomavirus (HPV) are known etiological factors for HNSCC [4, 5, 6]. Current clinical modalities include surgery, radiotherapy, cisplatin or paclitaxel chemotherapy, cetuximab‐targeted therapy, and pembrolizumab and nivolumab immunotherapy that blocks PD‐1 and PD‐L1 interactions [2, 7, 8, 9]. However, the overall response rate to these treatments is less than satisfactory, and the overall survival benefit remains low [10], particularly with recurrent/metastatic (R/M) HNSCC [1, 11].

Overexpression of epidermal growth factor receptor (EGFR) is one of the most common molecular alterations in HNSCC regardless of HPV status [12] and is a prognostic marker for this disease [13]. Targeting EGFR with either an antibody (e.g., cetuximab) or small molecule inhibitor (e.g., erlotinib) has been extensively investigated in clinical trials and has been approved by the Food and Drug Administration (FDA) for the treatment of patients with primary or R/M HNSCC [2, 11, 14, 15, 16]. However, clinical efficacy is modest and acquired resistance to EGFR inhibitors often occurs over time [1, 2, 17]. Thus, additional therapeutics with improved efficacy that could potentially overcome this resistance are urgently needed.

Immunotoxins or fusion toxins (ITs) combine cell surface binding ligands or antibody‐based single‐chain fragment variable (scFv) with a peptide toxin. In cancer treatment, the ligand or scFv binds to a cell surface receptor expressed or overexpressed by malignant cells, and the toxin triggers cell death [18]. The diphtheria toxin (DT)‐based monovalent human EGF fusion toxin (DAB_389_EGF) was first studied back in 1991 [19] and evaluated for treating human glioblastoma multiforme cells and non‐muscle‐invasive urinary bladder cancer [20, 21]. Further clinical development for systemic treatment was halted due to severe *in vivo* off‐target toxicities caused by the monovalent human EGF fusion toxin (mono‐EGF‐IT), despite its good *in vivo* efficacy against EGFR^+^ cancers. In this study, we developed a novel bivalent DT‐based EGF fusion toxin (bi‐EGF‐IT) using a unique DT‐resistant yeast *Pichia pastoris* expression system [22]. The bi‐EGF‐IT has an increased binding affinity *in vitro* and is more effective and less toxic at inhibiting primary HNSCC tumor growth and metastasis *in vivo* compared with mono‐EGF‐IT, representing a promising EGFR‐targeted drug candidate for HNSCC treatment.

## 2 Materials and methods

### 2.1 Cell lines, western blot, and antibodies

All HNSCC tumor cell lines, their clinical information, and source are listed in Table S1. The human EGFR^–^ tumor cell lines Jeko‐1 (ATCC® CRL‐3006™), Jurkat (clone E6‐1, ATCC® TIB‐152™), and EL4 (ATCC® TIB‐39™) were purchased from ATCC (Manassas, VA, USA). Western blot analysis was performed as described previously [23]. Briefly, protein samples were separated and transferred onto nitrocellulose membranes (Thermo Fisher Scientific, Waltham, MA, USA). The membranes were blocked and washed at room temperature with shaking. The fusion toxins were detected using mouse anti‐His Tag or anti‐DT primary antibodies and rat anti‐mouse IgG‐HRP secondary antibody. The proteins were detected using the TMB membrane peroxidase substrate (KPL Cat# 50‐77‐02). The antibodies used in this study are listed in Table S2.

### 2.2 Monovalent and bivalent EGF fusion toxin DNA constructs

**Fig. 1:**
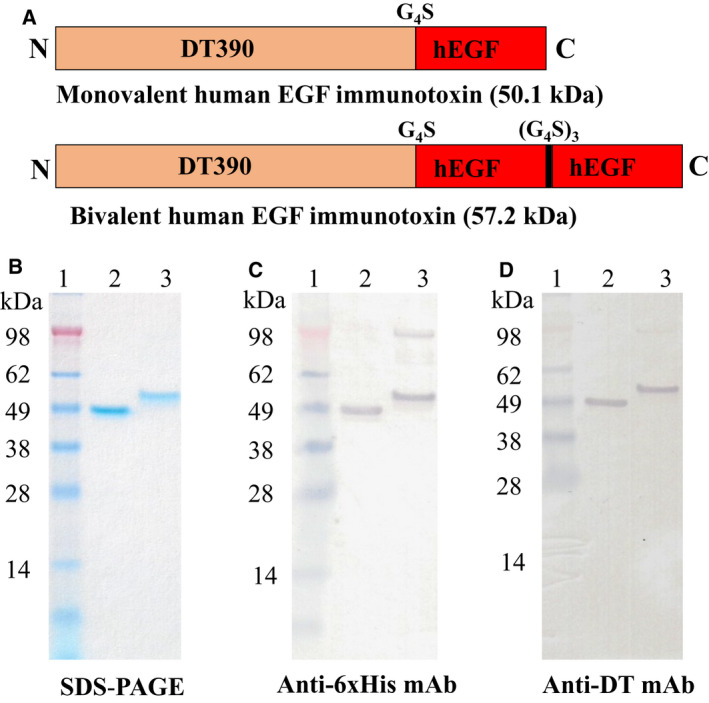
Schematic diagrams, SDS/PAGE, and western blot analysis of the mono‐EGF‐IT and bi‐EGF‐IT. (A) Schematic diagrams of the mono‐EGF‐IT and bi‐EGF‐IT. N, N‐terminal; C, C‐terminal. (B) SDS/PAGE (4–12% NuPAGE) of the mono‐EGF‐IT and bi‐EGF‐IT. (C) Western blot analysis using a mouse anti‐His mAb. (D) Western blot analysis using a mouse anti‐DT mAb. Lane 1: protein marker; lane 2: mono‐EGF‐IT (50.1 kDa); lane 3: bi‐EGF‐IT (57.2 kDa). The weak high molecular‐weight bands in lane 3 are dimers of bi‐EGF‐IT due to the formation of disulfide bonds.

As shown in Fig. 1, the human EGF (hEGF) fusion toxin contains two domains, DT390 [24] and human EGF ligand (UniProt P01133). A linker consisting of four glycines and a serine residue (G_4_S) connected the DT390 domain to the human EGF domain(s). The two human EGF domains of the bi‐EGF‐IT were joined by three tandem G_4_S linkers [(G_4_S)_3_]. Six histidines (6x His tag) were added to the C terminus of each construct to facilitate protein purification.

#### 2.2.1 Monovalent EGF fusion toxin DNA construct

Codon‐optimized hEGF DNA was synthesized by GenScript and cloned into the pwPICZalpha‐DT390 vector [25] between the *Nco*I and *Eco*RI sites, yielding the monovalent EGF fusion toxin DNA construct (mono‐EGF‐IT).

#### 2.2.2 Bivalent EGF fusion toxin DNA construct

To prepare the first hEGF insert, hEGF DNA was amplified using PCR primers for hEGF‐Nco carrying an *Nco*I site and hEGF‐Bam1 carrying a *Bam*HI site. The mono‐EGF‐IT DNA construct was used as the PCR template. The amplified PCR product was separated using DNA agarose gel electrophoresis. The expected PCR product band was cut out and extracted using the QIAquick Gel Extraction Kit (Qiagen, Germantown, MD, USA). The extracted DNA was digested using *Nco*I and *Bam*HI and cleaned using the QIAquick PCR Purification Kit (Qiagen), resulting in the first human EGF insert (Insert I). A similar approach was used to obtain the second hEGF insert (Insert II). The primers used to amplify the second hEGF DNA were hEGF‐Bam2 carrying a *Bam*HI site and hEGF‐Eco carrying an *Eco*RI site. The gel‐purified PCR product was digested using *Bam*HI and *Eco*RI and cleaned using the QIAquick PCR Purification Kit. Insert I and Insert II (*Nco*I‐EGF‐*Bam*HI‐EGF‐*EcoR*I) were cloned together into the pwPICZalpha‐DT390 vector between the *Nco*I and *Eco*RI to yield the bivalent EGF immunotoxin DNA construct (bi‐EGF‐IT), following sequencing confirmation. The PCR primers used in this study are listed in Table S3.

### 2.3 Protein expression

The Mono‐EGF‐IT and bi‐EGF IT DNA constructs were linearized and transformed into the DT‐resistant yeast *P. pastoris* strain [22] using the Gene Pulser Xcell Electroporation System (Bio‐Rad, Hercules, CA, USA). The transformed cells were spread on YPD agar plates (1% yeast extract, 2% peptone, 1.5% agar, 2% dextrose) containing 100 µg·mL^−1^ Zeocin and incubated at 30 °C for 3–4 days. Six colonies were randomly picked and cultured in 5 mL YPD at 30 °C for 24 h at 250 r.p.m. and then in YPG (1% yeast extract, 2% peptone, 1% glycerol) for another 24 h. The fusion toxin protein induction was carried out with 2 mL BMMYC (1% yeast extract, 2% peptone, 100 mm potassium phosphate, pH 7.0, 1.34% yeast nitrogen base without amino acids, 4 × 10^−5^% biotin, 0.5% methanol, and 1% casamino acids) for 48 h at 25 °C and 225 r.p.m. Methanol (0.5%) was added twice daily to maintain the methanol level. Antifoam (Emerald Performance Materials, Vancouver, WA, USA) was added to all growth and induction media at a concentration of 0.02%. PMSF, 1 mm; Sigma (St. Louis, MO, USA) was added to inhibit protein degradation during the induction phase. Penicillin (100 U·mL^−1^) and streptomycin (100 µg·mL^−1^) were added to all growth and induction media to inhibit bacterial contamination. The culture supernatants were analyzed using 4–12% SDS gels. One clone of each fusion toxin was selected for large‐scale expression. The Excella E24 incubator shaker (Eppendorf, Edison, NJ, USA) was used for large‐scale expression. The seed culture was prepared by inoculating a single colony into YPD medium and then incubating at 25 °C and 225 r.p.m. overnight. Next, 5% of the seed culture was transferred to 1‐L PYREX shake flasks containing 250 mL YPD medium and cultured at 30 °C and 250 r.p.m. for 24 h. The cells were centrifuged at 491 *g* for 5 min, and the cell pellet was resuspended in 250 mL YPG medium and cultured at 30 °C and 250 r.p.m. for 24 h. For the induction phase, cells were centrifuged at 1500 r.p.m. for 5 min, and the cell pellet was resuspended in 125 mL BMMYC induction medium and induced at 25 °C and 225 r.p.m. for 48 h. Methanol (0.5%) was added twice daily to maintain the methanol level. After the induction, the yeast cells were pelleted by centrifugation at 1692 *g* and 4 °C for 10 min. The supernatant was used for protein purification. Antifoam, PMSF, and penicillin/streptomycin were also added to the expression medium, as described for the small‐scale preparation.

### 2.4 Protein purification

Ni‐Sepharose™ 6 fast flow resin was used for the first step of the purification of the EGF fusion toxins. The resin was packed in an XK50 column (GE Healthcare, Chicago, IL, USA), equilibrated with 20 mm Tris/HCl pH 7.4, 0.5 m NaCl, and 5 mm imidazole. The sample was loaded onto the equilibrated column in 0.5 m NaCl, 20 mm Tris/HCl pH 7.4, and 5 mm imidazole. The column was washed with 20 mm Tris/HCl pH 7.4, 0.5 m NaCl, and 5 mm imidazole, and the bound proteins were eluted with 20 mm Tris/HCl pH 7.4, 0.5 m NaCl, and 500 mm imidazole. The purification fractions were analyzed using 4–12% SDS gels. The fractions containing the protein of interest were pooled and dialyzed using 3.5 kDa cutoff Spectra/Por membrane tubing (Spectrum Labs, Rancho Dominguez, CA, USA) against 20 mm Tris/HCl pH 8.0, 1 mm EDTA, and 5% glycerol at 4 °C with stirring. The dialysis buffer was replaced once. Strong anion exchange resin Poros 50 HQ (Applied Biosystems, Foster City, CA, USA) was packed in an XK16/20 column (GE Healthcare) for the second purification step. The column was equilibrated with 20 mm Tris/HCl pH 8.0, 1 mm EDTA, and 5% glycerol. The dialyzed sample was loaded onto the column and washed with 20 mm Tris/HCl pH 8.0, 1 mm EDTA, and 5% glycerol. The bound protein was eluted with 100 and 200 mm sodium borate, and then 200 mm sodium borate plus 50 mm NaCl (250 mm salt in total) in 20 mm Tris/HCl pH 8.0, 1 mm EDTA, and 5% glycerol. The purified fractions were analyzed using 4–12% SDS gels. The fractions containing the protein of interest were pooled and dialyzed using the 3.5 kDa cutoff Spectra/Por membrane tubing against PBS plus 5% glycerol at 4 °C with stirring. The dialysis buffer was replaced once. Protein concentration was measured using the Pierce BCA Protein Assay Kit (Thermo Fisher Scientific). DT390 and anti‐murine PD‐1 immunotoxin (mPD1‐IT) were also expressed and purified using the same DT‐resistant yeast *P. pastoris* expression system.

### 2.5 Biotin labeling of the EGF fusion toxins

EGF fusion toxins were labeled with EZ‐Link Sulfo‐NHS‐Biotin (Thermo Fisher Scientific). One milligram of NHS‐Biotin was added to one milligram of monovalent or bivalent EGF fusion toxin. The solution was incubated for 2 h at 4 °C with rocking. The samples were transferred to a Slide‐A‐Lyzer dialysis cassette (10K MWCO, 0.5–3 mL; Thermo Fisher) and dialyzed against 1× PBS for 24 h at 4 °C with stirring. The dialysis buffer was replaced once.

### 2.6 Binding affinity of EGF fusion toxins to EGFR^+^ HNSCC cells

EGFR^+^ HNSCC cells were stained with biotinylated mono‐EGF‐IT or bi‐EGF‐IT at a range of concentrations (0.01–200 nm). The biotin‐labeled anti‐human EGFR monoclonal antibody (mAb) (Cat #555997; BD PharMingen, San Jose, CA, USA) was used as a positive control at a final concentration of 36 nm. Negative control cells were stained only with streptavidin (SA)‐PE, at a final concentration of 1.5 ng·µL^−1^. Flow cytometry was carried out using a CytoFLEX Flow Cytometer (Beckman Coulter, Indianapolis, IN, USA), and data were analyzed using flowjo software (FlowJo; LLC, Ashland, OR, USA). Biotinylated anti‐murine PD1 immunotoxin was included as biotinylated protein control.

### 2.7 Blocking of the binding of anti‐human EGFR mAb to HNSCC cells by EGF fusion toxins

Cal27 HNSCC cells were resuspended at a concentration of 1 × 10^7^ cells per mL in FACS medium and aliquoted into FACS tubes at 1 × 10^6^ cells/tube. Nonbiotinylated mono‐EGF‐IT or bi‐EGF‐IT was added to the cells at a range of concentrations (0.08, 0.8, 8, 80, 800, and 1600 nm), and the cells were incubated for 5 min at 4 °C in the dark. Biotin‐labeled anti‐human EGFR mAb (Cat #555997; BD Pharmingen) was then added to each tube at a final concentration of 0.36 nm, and the cell suspensions were incubated at 4 °C in the dark for 30 min. The cells were washed twice with FACS medium (2 mL) and centrifuged at 1200 r.p.m. for 5 min at 4 °C. SA‐PE was added to each tube at a final concentration of 1.5 ng·µL^−1^, and the cells were incubated in the dark at 4 °C for 15 min. The cells were then washed once and then resuspended in 500 μL FACS medium. FACS analysis was carried out using a CytoFLEX Flow Cytometer (Beckman Coulter), and data were analyzed using flowjo software (FlowJo; LLC).

### 2.8 *K*_D_ determination

Binding of mono‐EGF‐IT or bi‐EGF‐IT to EGFR^+^ HNSCC cells was performed using a wide biotinylated mono‐EGF‐IT or bi‐EGF‐IT concentration range. *K*_D_ determination was performed on the flow cytometry data using nonlinear regression with the saturation binding equation (graphpad prism 9.0.0). The mean fluorescence intensity (MFI) was plotted versus the biotinylated mono‐EGF‐IT or bi‐EGF‐IT concentrations. Nonlinear regression was based on the equation *Y* = *B*_max_ × *X*/(*K*_D_ + *X*), where *Y* = MFI at the given biotinylated fusion toxin concentration after subtracting the background, *X* = biotinylated fusion toxin concentration, and *B*_max_ = the maximum specific binding in the same units as *Y*.

### 2.9 *In vitro* efficacy

The *in vitro* efficacy of mono‐EGF‐IT and bi‐EGF‐IT was determined in 14 human HNSCC tumor cell lines using the CellTiter‐Glo® Luminescent Cell Viability Assay (Promega, Madison, WI, USA), as described previously [26]. This assay measures the luminescence produced by adenosine triphosphate (ATP) production from metabolically active cells. Increasing concentrations of mono‐EGF‐IT and bi‐EGF‐IT cause cell death and a corresponding reduction in ATP‐related fluorescence. The luminescence signals were recorded using a BioTek Synergy LX Multi‐Mode Reader. The EGFR inhibitor erlotinib and mPD1‐IT were used as positive and negative controls, respectively.

### 2.10 *In vivo* efficacy

The NOD/SCID IL‐2 receptor γ^−/−^ *(NSG*) mouse breeding pairs were purchased from Jackson Laboratories (Bar Harbor, ME, USA) and bred in the animal facility of University of Colorado Anschutz Medical Campus. The experiments described were approved by the University of Colorado Anschutz Medical Campus Animal Care and Use Committee (IACUC). *NSG* mice (6–8 weeks) were divided into four treatment groups: (a) DT390, negative control; (b) mono‐EGF‐IT; (c) bi‐EGF‐IT; and (d) erlotinib, positive control at a dose reported previously [27, 28].

Three complementary HNSCC mouse models were used to evaluate *in vivo* efficacy: subcutaneous xenografts, orthotopic tongue xenografts, and experimental metastasis. For the subcutaneous xenograft model, human Cal27 HNSCC tumor cells (8 × 10^6^ cells) were subcutaneously injected into the right flank. In the orthotopic tongue xenograft model, *NSG* mice were anesthetized with isoflurane, and Cal27 cells [8 × 10^6^ cells in 50 µL Dulbecco's Modified Eagle's medium (DMEM)] were injected into the tongue. Finally, for the experimental metastasis model, Cal27 cells (1 × 10^6^ cells in 200 µL DMEM) were injected intravenously via the tail vein. For all three models, the tumor‐bearing mice were randomly divided into the four treatment groups on day 3 postinoculation.

On day 4 postinoculation, treatment commenced for all three HNSCC models. Mono‐EGF‐IT, bi‐EGF‐IT, and DT390 were administered by intraperitoneal (IP) injection at a dose of 50 µg·kg^−1^. Erlotinib was administered via intragastric gavage at a dose of 20 mg·kg^−1^. All treatments were administered once daily for 10 consecutive days. The animals were observed daily for signs and symptoms of illness, and tumors were measured using digital vernier calipers every 3 days, as previously described [23, 25, 29, 30]. Mice were euthanized at their end point defined as when the tumor size became greater than 1 cm^3^, or body weight loss was greater than 15%. The tumor volume was calculated according to the formula: volume (mm^3^) = [length × (width × 2)]/2. Necropsy was performed by the veterinarian pathologist on mice receiving mono‐EGF‐IT and bi‐EGF‐IT to evaluate toxicity.

### 2.11 Statistical analysis

The half‐maximal inhibitory concentration (IC_50_s) were determined using nonlinear regression (graphpad prism 9.0.0, GraphPad Software; San Diego, CA, USA). The *P*‐values for the survival curves were calculated using the Mantel–Cox log‐rank test (graphpad prism 9.0.0). Because the distribution of the *K*_D_ values from all the cell lines was skewed, the nonparametric Wilcoxon signed‐rank test was carried out to test the null hypothesis of no difference between the *K*_D_ values from the mono‐EGF‐IT and bi‐EGF‐IT groups. The *P*‐values for other comparisons were calculated using the two‐tailed Student *t*‐test (graphpad prism 9.0.0). *P* < 0.05 was considered statistically significant.

## 3 Results

### 3.1 Construction and expression of monovalent and bivalent human EGF fusion toxins

Codon‐optimized human EGF (hEGF) DNA was synthesized and cloned into the truncated DT‐containing expression vector pwPICZalpha‐DT390 (Fig. 1A), as previously described [31]. Both the monovalent human EGF fusion toxin (mono‐EGF‐IT) and the bivalent human EGF fusion toxin (bi‐EGF‐IT) were expressed and purified using a unique DT‐resistant yeast *P. pastoris* expression system [22, 31]. The final purification yields were ~ 14 and 10 mg per liter of harvested supernatant for mono‐EGF‐IT and bi‐EGF‐IT, respectively. The purified mono‐EGF‐IT and bi‐EGF‐IT were analyzed using SDS/PAGE and western blot. The expected molecular weights of 50.1 and 57.2 kDa were demonstrated for mono‐EGF‐IT and bi‐EGF‐IT, respectively (Fig. 1B‐D).

### 3.2 *In vitro* binding affinity of mono‐EGF‐IT and bi‐EGF‐IT to HNSCC cell lines

**Fig. 2:**
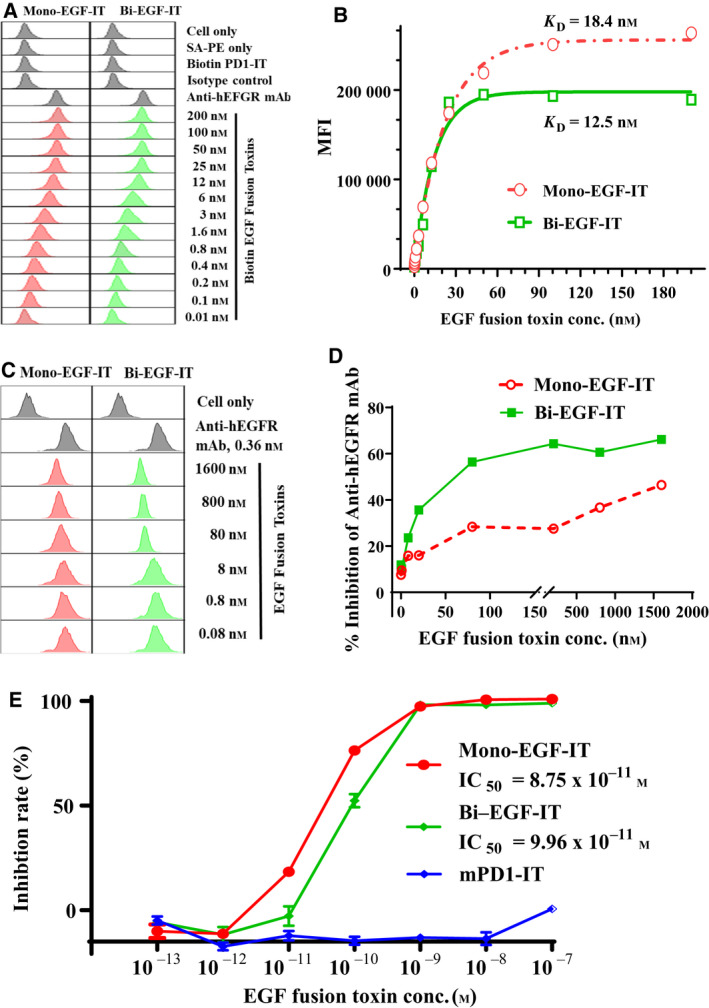
*In vitro* binding affinity and efficacy analysis of the mono‐EGF‐IT and bi‐EGF‐IT to the human EGFR^+^ HNSCC cell line. (A) Binding affinity analysis of the mono‐EGF‐IT and bi‐EGF‐IT to the human EGFR^+^ HNSCC cell line, Cal27, using flow cytometry. Anti‐human EGFR mAb was used as a positive control, and biotinylated anti‐murine PD‐1 immunotoxin served as a negative background control for protein biotinylation. The data are representative of three individual experiments. (B) *K*_D_ determination of the human EGF fusion toxins for Cal27 cells using flow cytometry and nonlinear least‐squares fitting. The MFI was plotted over a wide range of biotinylated mono‐EGF‐IT or bi‐EGF‐IT concentrations. Nonlinear regression was based on the equation *Y* = *B*_max_ × *X*/(*K*_D_ + *X*), where *Y* = MFI at the given biotinylated fusion toxin concentration after subtracting the background, *X* = biotinylated fusion toxin concentration, and *B*_max_ = the maximum specific binding in the same units as *Y*. (C) Analysis of the blocking of anti‐human EGFR mAb binding to the human EGFR^+^ HNSCC cell line, Cal27, by mono‐EGF‐IT and bi‐EGF‐IT using flow cytometry. (D) The percentage inhibition of anti‐human EGFR mAb binding to Cal27 cells is plotted versus the concentration of binding competitor (mono‐EGF‐IT or bi‐EGF‐IT). The data are representative of three individual experiments. (E) *In vitro* efficacy of the mono‐EGF‐IT and bi‐EGF‐IT in the human EGFR^+^ HNSCC cell line, Cal27 determined by the CellTiter‐Glo® Luminescent Cell Viability Assay (red line: mono‐EGF‐IT group; green line: bi‐EGF‐IT group; blue line: anti‐murine PD‐1 immunotoxin group as the negative control). Y‐axis: percent inhibition of cell viability determined by the number of viable cells based on the quantification of ATP. X‐axis: fusion toxin concentration. Cycloheximide (1.25 mg·mL^−1^) was used as a positive control. The negative control wells contained cells without fusion toxin. Data are from three individual experiments. Error bars indicate SD.

The binding affinities of the two fusion toxins were evaluated in 14 HNSCC cell lines that represent HNSCC tumors of different anatomic locations, staging, HPV, and mutation status (Table S1). EGFR expression was confirmed in all 14 HNSCC cell lines by flow cytometry (Figs 2A,B and S1). As shown in Fig. 2A,B, biotinylated mono‐EGF‐IT and bi‐EGF‐IT bound to human Cal27 HNSCC cells in a dose‐dependent fashion, with a *K*_D_ of 18.4 nm for mono‐EGF‐IT and 12.5 nm for bi‐EGF‐IT [32]. One limitation to the flow cytometry binding assay was that the extent of biotinylation should be similar between the constructs. Otherwise, it could be hard to attribute the differences in fluorescence intensities to a change in affinity. To overcome this limitation, we performed a blocking assay using nonbiotinylated mono‐EGF‐IT or bi‐EGF‐IT to block the binding of the anti‐human EGFR mAb to the EGFR^+^ HNSCC cell line, Cal27 [33]. The results confirmed that bi‐EGF‐IT bound significantly stronger to Cal27 cells compared with mono‐EGF‐IT (Fig. 2C,D). Both mono‐EGF‐IT and bi‐EGF‐IT bound to the remaining 13 HNSCC cell lines in a dose‐dependent manner (Fig. S1). The *K*_D_ values for mono‐EGF‐IT and bi‐EGF‐IT obtained for each cell line are summarized in Table 1. Of note, the mean *K*_D_ for bi‐EGF‐IT (5.05 nm) was significantly lower than that for mono‐EGF‐IT (12.21 nm), indicating that the former had a better binding affinity (Fig. S2).

**Table 1:** Human EGF fusion toxin *K*_D_s for human HNSCC cell lines.

	Mono‐EGF‐IT (nm)	Bi‐EGF‐IT (nm)
Cal27	18.44	12.45
FaDu	7.13	6.20
HN6	14.71	3.44
UMSCC‐1	4.68	1.30
UMSCC‐2	10.95	8.93
UMSCC‐10A	3.87	3.63
UMSCC‐10B	9.58	1.34
UMSCC‐11A	2.55	1.12
UMSCC‐22A	4.81	3.40
UMSCC‐22B	7.34	3.41
UMSCC‐47	36.73	11.08
Tul67	15.70	5.30
Vul131	33.43	7.51
Vul365	0.98	1.54

### 3.3 *In vitro* efficacy of mono‐EGF‐IT and bi‐EGF‐IT against HNSCC cell lines

The *in vitro* efficacy of mono‐EGF‐IT and bi‐EGF‐IT against HNSCC cell lines was assessed using the CellTiter‐Glo® Luminescent Cell Viability Assay. Both mono‐EGF‐IT and bi‐EGF‐IT effectively inhibited Cal27 HNSCC cell growth with IC_50_ values of 8.75 × 10^−11^ m and 9.96 × 10^−11^ m for mono‐EGF‐IT and bi‐EGF‐IT, respectively (Fig. 2E). The effectiveness of both fusion toxins in inhibiting the other 13 HNSCC cell lines is shown in Fig. S3. The inhibition of the 14 HNSCC cell lines by erlotinib is shown in Fig. S4. The IC_50_ values for mono‐EGF‐IT, bi‐EGF‐IT, and erlotinib in each cell line are presented in Table 2.

**Table 2:** Human EGF fusion toxin IC_50_s for HNSCC cell lines.

	Mono‐EGF‐IT (M)	Bi‐EGF‐IT (M)	Erlotinib (μm)
Cal27	8.75E‐11	9.96E‐11	1.87
FaDu	8.81E‐11	3.58E‐11	2.83
HN6	5.17E‐11	1.49E‐10	0.94
UMSCC‐1	4.6E‐11	8.48E‐10	2.46
UMSCC‐2	2.32E‐11	1.1E‐10	1.59
UMSCC‐10A	9.77E‐10	6.37E‐10	15.16
UMSCC‐10B	1.59E‐09	4.29E‐10	3.81
UMSCC‐11A	3.36E‐12	1.48E‐11	2.76
UMSCC‐22A	2.72E‐10	4.87E‐08	0.16
UMSCC‐22B	5.17E‐10	4.54E‐09	1.74
UMSCC‐47	4.95E‐11	2.3E‐10	4.12
Tul67	4.82E‐11	4.57E‐10	0.41
Vu1131	1.8E‐11	2.93E‐11	0.33
Vu1365	7.85E‐10	9.21E‐09	1.54

**Fig. 3:**
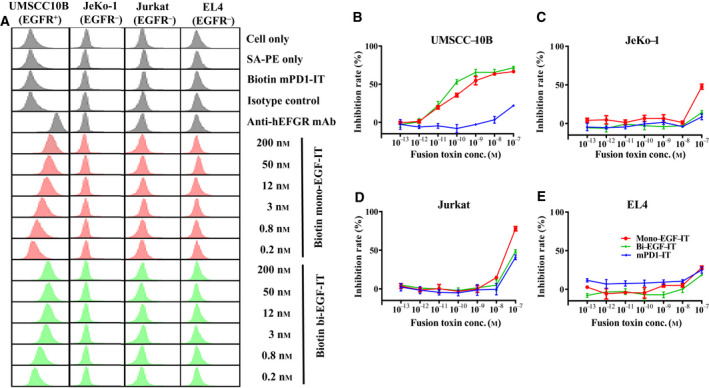
Off‐target analysis of the human EGF fusion toxins using three human EGFR^–^ tumor cell lines (JeKo‐1, Jurkat, and EL4). The human EGFR^+^ HNSCC cell line, UMSCC10B, was included as a positive control. (A) Binding affinity analysis of the human EGF fusion toxins to three human EGFR^–^ tumor cell lines using flow cytometry. The data are representative of three individual experiments. (B‐E) *In vitro* efficacy of human EGF fusion toxins in three human EGFR^–^ and one EGFR^+^ tumor cell lines using the CellTiter‐Glo® Luminescent Cell Viability Assay. (B) UMSCC‐10B (EGFR^+^). (C) Jeko‐1 (EGFR^−^). (D) Jurkat (EGFR^−^). (E) EL4 (EGFR^−^). Data are from three individual experiments. Error bars indicate SD.

We also performed *in vitro* off‐target analysis using three human EGFR^–^ tumor cell lines (JeKo‐1, Jurkat, and EL‐4). There was no *in vitro* binding of either mono‐EGF‐IT or bi‐EGF‐IT to these three cell lines compared with the EGFR‐positive UMSCC‐10B cell line (Fig. 3A). Likewise, neither fusion toxin affected the viability of the three EGFR^–^ human cancer cell lines up to 10^−8^ m concentration. Nonspecific toxicity was demonstrated at the concentration of 10^−7^ m (Fig. 3B–E).

### 3.4 *In vivo* efficacy of mono‐EGF‐IT and bi‐EGF‐IT against HNSCC mouse models

**Fig. 4:**
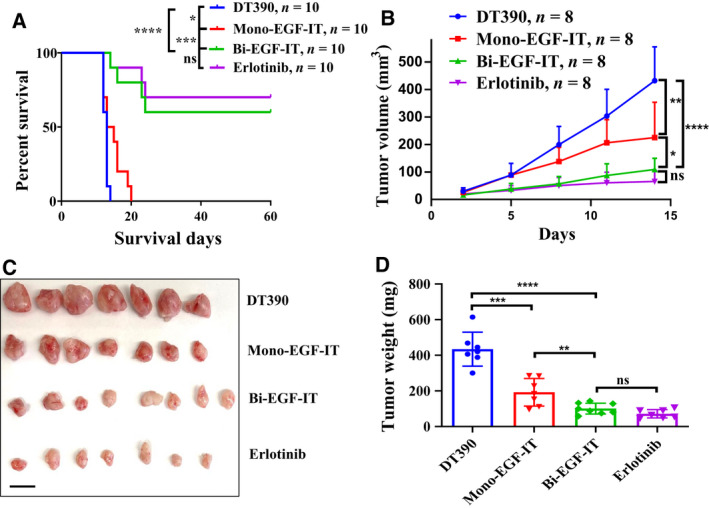
*In vivo* efficacy of human EGF fusion toxins against subcutaneous xenografts in *NSG* mice. (A) Cal27 cells were subcutaneously injected into the right flank and treated with DT390 (*n* = 10), mono‐EGF‐IT (*n* = 10), bi‐EGF‐IT (*n* = 10), or erlotinib (*n* = 10) once daily for 10 consecutive days beginning on day 4 after the tumor cell injection. Kaplan–Meier survival curves were recorded for the DT390 (blue line), mono‐EGF‐IT (red line), bi‐EGF‐IT (green line), and erlotinib (purple line) groups. (B‐D) Cal27 cells were subcutaneously injected into the flanks of a second cohort of *NSG* mice that were then treated with DT390 (*n* = 8), mono‐EGF‐IT (*n* = 8), bi‐EGF‐IT (*n* = 8), or erlotinib (*n* = 8). Mice were euthanized on day 14 after tumor cell injection when the first mouse in the DT390 group reached the end point. (B) Tumor volumes were measured periodically, and the growth kinetics of the four groups were plotted. (C) Image of harvested tumors on day 14 and (D) the mean tumor weight for each group. Scale bar: 1 cm. *P < 0.05; **P < 0.01; ***P < 0.001; ****P < 0.0001; ns: not significant. The *P*‐values for the survival curves in panel A were calculated using the Mantel–Cox log‐rank test and that for the comparisons in panels B and D were calculated using the two‐tailed Student *t*‐test (graphpad prism 9.0.0).

The *in vivo* efficacy of mono‐EGF‐IT and bi‐EGF‐IT against HNSCC was assessed using three cancer mouse models: subcutaneous xenografts, orthotopic xenografts in the mouse tongue, and experimental metastasis. In the first model, we subcutaneously (SQ) injected Cal27 HNSCC tumor cells into the right flank of NSG mice. Beginning 4 days postinoculation, mice were treated with 50 µg·kg^−1^ mono‐EGF‐IT or bi‐EGF‐IT by IP injection or 20 mg·kg^−1^ erlotinib by intragastric gavage daily for 10 consecutive days. Remarkably, bi‐EGF‐IT increased the median survival time of tumor‐bearing animals from 14 days (DT390‐negative control group) to at least 60 days (*P* < 0.0001) (all mice in the bi‐EGF‐IT and erlotinib groups were euthanized at day 60 requested by the IACUC due to over the limits of end points in our animal protocol). The median survival time for the bi‐EGF‐IT group was comparable to that of the erlotinib group (Fig. 4A). In contrast, mono‐EGF‐IT did not significantly prolong the median survival time (15 versus 14 days). The reductions in tumor volume, weight, and size for the bi‐EGF‐IT group (Fig. 4B‐D) were consistent with the prolonged median survival time. Indeed, bi‐EGF‐IT significantly decreased tumor size and weight by ~ 80%.

**Fig. 5:**
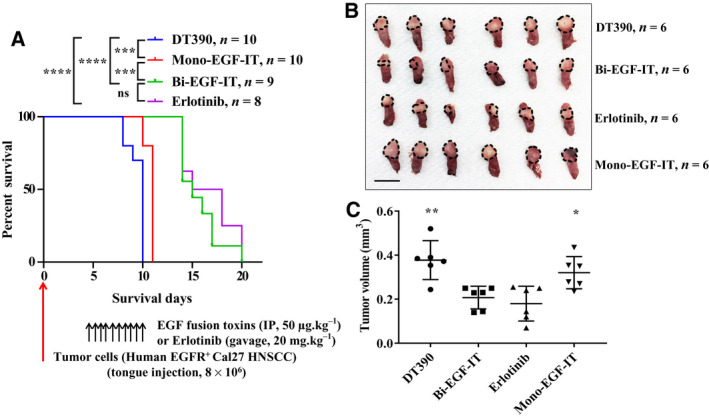
*In vivo* efficacy of EGF fusion toxins against tongue SCCs in *NSG* mice. (A) Cal27 cells were injected into the tongues of *NSG* mice and treated with DT390 (*n* = 10), mono‐EGF‐IT (*n* = 10), bi‐EGF‐IT (*n* = 9), or erlotinib (*n* = 8) once daily for 10 consecutive days starting on day 4 after the tumor cell injection. Kaplan–Meier survival curves were recorded for the DT390 (blue line), mono‐EGF‐IT (red line), bi‐EGF‐IT (green line), and erlotinib (purple line) groups. The timeline and detailed schedules for tumor cell injection and treatments are shown under the survival curve. The vertical arrows indicate the days on which the tumor cells or the treatments were administered. (B‐C) Cal27 cells were injected into the tongues of a second cohort of *NSG* mice that were then treated with DT390 (*n* = 6), mono‐EGF‐IT (*n* = 6), bi‐EGF‐IT (*n* = 6), or erlotinib (*n* = 6). The mice were euthanized on day 8 after tumor cell injection when the first mouse in the DT390 group reached the end point. (B) Image of tongue SCCs (circled by the black dotted lines) and (C) tumor volume comparison of the treatment groups. Scale bar: 1 cm. *P < 0.05; **P < 0.01; ***P < 0.001; ****P < 0.0001; ns: not significant. The *P*‐values for the survival curves in panel A were calculated using the Mantel‐Cox log‐rank test and that for the comparisons in panel C were calculated using the two‐tailed Student *t*‐test (graphpad prism 9.0.0).

To better mimic the clinical treatment of HNSCC, we used an orthotopic model of tongue SCC to assess the *in vivo* effectiveness of mono‐EGF‐IT and bi‐EGF‐IT. We injected Cal27 cells into the tongues of NSG mice. Beginning 4 days postinoculation, mice were treated with 50 µg·kg^−1^ mono‐EGF‐IT or bi‐EGF‐IT by IP injection or 20 mg·kg^−1^ erlotinib by intragastric gavage daily for 10 consecutive days. Because tongue tumors affect the ability of mice to eat and drink, mice were euthanized at an earlier end point than with the subcutaneous tumor model. Therefore, the median survival time prolongation was shorter in this model. As shown in Fig. 5A, bi‐EGF‐IT significantly increased the median survival time of mice with tongue tumors from 10 days (DT390‐negative control group) to 15 days (*P* < 0.0001). To further characterize the effect of the EGF fusion toxins on tumor volume, we repeated the orthotopic tongue SCC model study and euthanized the mice 8 days after tumor cell inoculation. As shown in Fig. 5B,C, the tongue SCCs were significantly smaller in both the bi‐EGF‐IT and erlotinib groups than those from the mono‐EGF‐IT and DT390 control groups.

**Fig. 6:**
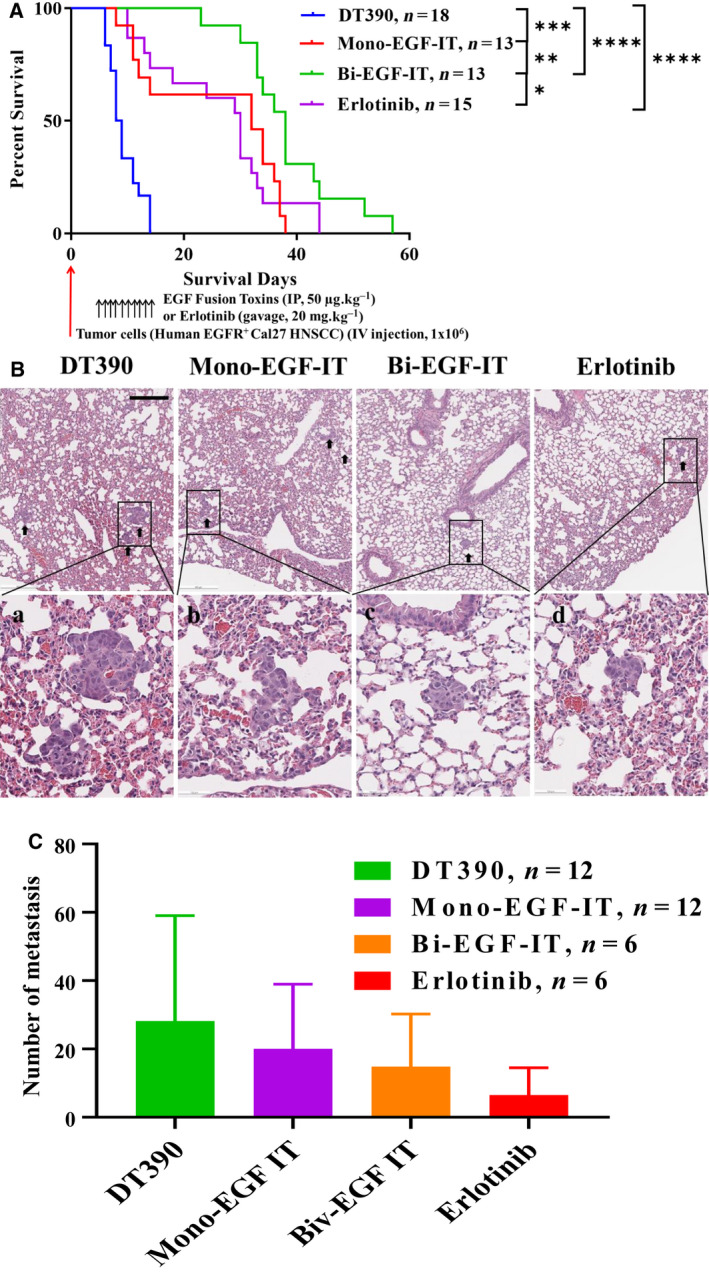
*In vivo* efficacy of EGF fusion toxins against an experimental lung metastasis mouse model. (A) Cal27 cells were intravenously injected into *NSG* mice that were then treated with DT390 (*n* = 18), mono‐EGF‐IT (*n* = 13), bi‐EGF‐IT (*n* = 13), or erlotinib (*n* = 15) once daily for 10 consecutive days starting on day 4 after the tumor cell injection. Kaplan–Meier survival curves were recorded for the DT390 (blue line), mono‐EGF‐IT (red line), bi‐EGF‐IT (green line), and erlotinib (purple line) groups. The timeline and detailed schedules for tumor cell injection and treatments are shown under the survival curve. The vertical arrows indicate the days on which the tumor cells or the treatments were administered. *P < 0.05; **P < 0.01; ***P < 0.001; ****P < 0.0001. The *P*‐values for the survival curves in panel A were calculated using the Mantel–Cox log‐rank test (graphpad prism 9.0.0). (B‐C) Cal27 cells were intravenously injected into a second cohort of *NSG* mice that were then treated with DT390 (*n* = 12), mono‐EGF‐IT (*n* = 12), bi‐EGF‐IT (*n* = 6), or erlotinib (*n* = 6). The mice were euthanized on day 10 after tumor cell injection when the first mouse in the DT390 group reached the end point. (B) H&E pictures of lung metastases (black arrowheads, upper panels), with higher magnification pictures of the black rectangles (lower panels). (C) The numbers of lung metastasis present in the DT390, mono‐EGF‐IT, bi‐EGF‐IT, and erlotinib treatment groups. Scale bar: 500 µm. Error bars indicate SD.

For the experimental metastasis model, we intravenously injected Cal27 cells into *NSG* mice. Beginning 4 days postinoculation, mice were treated with 50 µg·kg^−1^ mono‐EGF‐IT or bi‐EGF‐IT by IP injection or 20 mg·kg^−1^ erlotinib by intragastric gavage daily for 10 consecutive days. As shown in Fig. 6A, all treatments significantly prolonged the median survival time (i.e., 8.5 days for the DT390‐negative control group versus 32, 38, and 30 days for the mono‐EGF‐IT, bi‐EGF‐IT, and erlotinib groups, respectively). To further assess the effects of the fusion toxins on metastasis, we repeated the experimental metastasis study and euthanized all mice 10 days after tumor cell inoculation. As shown in Fig. 6B,C, the numbers of metastases in the lungs of mice treated with mono‐EGF‐IT, bi‐EGF‐IT, or erlotinib were reduced compared with the DT390‐negative control group. This reduction was greater in the bi‐EGF‐IT group than in the mono‐EGF‐IT group. The median survival times for the mice in the different treatment groups in the three mouse models are summarized in Table 3.

**Table 3:** Median survival time for tumor‐bearing *NSG* mice.

Tumor model	Median survival time (Days)			
	DT390	Mono‐EGF‐ITT	Bi‐EGF‐IT	Erlotinib
Subcutaneous xenograft model	13	14	> 60	> 60
Tongue orthotopic model	10	11	15	16.5
Lung metastasis model	8.5	32	38	30

Mice treated with mono‐EGF‐IT appeared generally unhealthy and lethargic and developed skin rashes. These adverse effects were not observed with bi‐EGF‐IT. To further assess these toxic effects, we performed an *in vivo* toxicity study in non‐tumor‐bearing mice. Mice treated with mono‐EGF‐IT, but not bi‐EGF‐IT, were again generally unhealthy and lethargic with skin rashes (Fig. S5A). Necropsy of the mono‐EGF‐IT‐treated mice 13 days after the first drug injection showed diffuse pale livers with moderate reticular patterns, suggesting lobular congestion or necrosis (Fig. S5B). In contrast, no significant abnormal findings were observed for bi‐EGF‐IT‐treated mice. Similarly, the entire gastrointestinal tract was mildly pale with scant ingesta in the stomach or cecum in the mono‐EGF‐IT group compared with the normal appearance of the gastrointestinal tract in the bi‐EGF‐IT group (Fig. S5C). These data demonstrated that bi‐EGF‐IT had markedly less *in vivo* off‐target toxicities than the monovalent EGF fusion toxin.

## 4 Discussion

It is always a challenge to express recombinant immunotoxins and fusion toxins using an *E. coli* expression system. The FDA‐approved Ontak® was discontinued due to low purification quality related to the *E. coli* expression system. Our bi‐EGF‐IT was produced using a unique DT‐resistant *P. pastoris* yeast expression system with high yield and excellent quality [25], which will facilitate future clinical development and success in the market. The DT‐based monovalent EGF fusion toxin, DAB_389_EGF, was first studied by Shaw *et al*. [19]. It was subsequently investigated for the treatment of human glioblastoma multiforme [20] and non‐muscle‐invasive urinary bladder cancer [21]. An antibody‐based bivalent immunotoxin against EGFR has been studied using an HNSCC mouse model [34]. Compared to these previous works, the compelling highlight of the current study is that we discovered that the bi‐EGF‐IT molecule had improved efficacy and markedly less *in vivo* off‐target toxicity than its monovalent counterpart (mono‐EGF‐IT). Bi‐EGF‐IT is an endogenous ligand EGF‐based bivalent fusion toxin rather than an antibody fragment (scFv)‐based bivalent immunotoxin [34]. Although we did not do a side‐by‐side comparison of our ligand‐based bi‐EGF‐IT vs the antibody‐based bivalent immunotoxin in this study, it seems that our EGF‐based bivalent fusion toxin may be more potent based on an IC_50_ comparison (bi‐EGF‐IT IC_50_ range: 0.029–48.7 nm; antibody‐based bivalent immunotoxin IC_50_ range: 0.24–156 nm [34]). No immunogenicity is also an advantage of an endogenous ligand over antibody fragment for construction of the fusion toxins.

We are well aware of the limitation of the *in vitro* binding model used in this study, which does not account for the possible binding of the two moieties of the bi‐EGF‐IT. Additional validations using methods, such as surface plasmon resonance system or biolayer interferometry, would be more confirmative approaches for the binding affinity comparison between the mono‐EGF‐IT vs bi‐EGF‐IT to the recombinant human EGFR protein.

Immunogenicity is a common concern for multiple courses of immunotoxin and fusion toxin treatment. Because we used immunodeficient *NSG* mice in this study, we could not assess the immunogenicity. We predict that bi‐EGF‐IT will also induce neutralizing antibodies based on our previous monkey study data [35]. However, DT‐based IL‐2 fusion toxin Ontak® and IL‐3 fusion toxin ELZONRIS™ were approved by FDA for multiple treatment courses. We speculate that our bi‐EGF‐IT could also be administered for multiple courses as the same truncated DT DT390 was used. Of note, the DT390 amino acid sequence used in this study include an extra alanine in the N terminus and two N‐linked glycosylation site mutations (S18A and N235A) [36]. Immunogenicity of DT390‐based immunotoxin can be reduced by depleting the immunogenetic B‐ and T‐cell epitopes of DT390 domain as reported by Mazor and Pastan 2020 [37].

Interestingly, the superiority of bi‐EGFR‐IT over mono‐EGFR‐IT was not observed in the *in vitro* cell viability assay but was clearly demonstrated *in vivo*, which may be due to the increased binding affinity of bi‐EGF‐IT versus mono‐EGF‐IT. The discrepancy between the cell viability assay and *in vivo* efficacy data demonstrates the limitations to performing efficacy studies in the culture dish and emphasizes the value of evaluating efficacy by *in vivo* approaches. In this study, we used three different Cal 27 HNSCC models *in vivo*. Cal27 cells were selected for *in vivo* testing because they overexpress EGFR, and these cells have been widely used in the field and characterized as an orthotopic model. We are well aware of the limitations of using immunocompromised mice to mimic HNSCC development and metastasis. Syngeneic murine HNSCC mouse models will provide a complementary approach. In the future, a humanized HNSCC mouse model might be the best choice for evaluating potential immunotoxin therapy.

The main side effects of targeting EGFR are caused by off‐target effects because EGFR is expressed in many healthy tissues, including the skin, liver, and gastrointestinal tract [38]. Hence, targeting EGFR often results in several adverse effects, including skin rashes, diarrhea, and lethargy. In this study, we observed these adverse side effects in mono‐EGF‐IT‐treated mice. In addition, our ongoing experiments have demonstrated that the human EGF fusion toxins can cross‐react with murine EGFR^+^ tumor cells in a syngeneic mouse tumor model. These data suggest that human EGF fusion toxins could also react with healthy mouse host cells expressing EGFR. Toxicity caused by the off‐target effects has been the major obstacle preventing EGF fusion toxins (e.g., DAB_398_EGF) from moving forward into clinical use. However, these off‐target and adverse effects were markedly less following bi‐EGF‐IT treatment. Although we do not understand the underlying mechanism, the lower toxicity in combination with better efficacy observed with bi‐EGF‐IT *in vivo* provides a better therapeutic window for its potential clinical development, not only for the treatment of primary tumors but also for recurrent and metastatic disease.

Targeting EGFR is the major cornerstone for targeted HNSCC therapy [1, 2]. EGFR‐targeted therapies, such as cetuximab, competitively block the binding of endogenous EGF ligand to the EGFR, which blocks the receptor‐dependent signal transduction pathways for the growth and survival of tumor cells [15]. However, resistance to EGFR inhibitors is a challenge for achieving clinical benefit and remains one of the hottest topics for developing novel targeted EGFR therapy [1, 2, 14]. Acquired mutations or compensatory activation of downstream pathways (e.g., PI3K, PTEN, Met, or Ras) are some of the main resistance mechanisms for EGFR inhibitors used in HNSCC treatment [15]. Although speculative, the bi‐EGF‐IT molecule may overcome these resistance mechanisms, given that bi‐EGF‐IT directly inhibits protein synthesis to induce cell death independent of the crosstalk between EGFR signaling pathways. Bi‐EGF‐IT exerts its function as long as it is bound to EGFR and is internalized [39]. Given the cytotoxic mechanism of immunotoxin‐based EGF targeted therapy is markedly distinct from that of EGFR‐targeted tyrosine kinase inhibitor, such as erlotinib, we would expect the bi‐EGF‐IT will overcome resistance commonly occurred in tyrosine kinase inhibitor‐based therapy. One good example has been shown in an EGF fusion toxin with gelonin, which exhibits potent efficacy in several erlotinib and cetuximab‐resistant HNSCC cell lines [40].

Clinically approved immunotherapy using immune checkpoint inhibitors (e.g., pembrolizumab or nivolumab) represents a major change in the treatment of HNSCC patients [2]. However, the effective rate for this therapy is around 20% [1, 7]. Exploring combination therapeutics and identifying biomarkers for patient stratification are expected to improve the effectiveness of this treatment [7]. To this point, oncogene‐targeted drug‐induced innate immune signaling may provide an opportunity for combination immunotherapy [41]. In particular, cancer cells undergo reprogramming of the immune landscape upon inhibition of receptor tyrosine kinases, which provides the rationale for combining current bi‐EGF‐IT therapy with immunotherapy [42]. We are currently testing this hypothesis by investigating the immune landscape upon bi‐EGF‐IT treatment using a syngeneic murine HNSCC mouse model.

For our next steps, we will investigate the efficacy of bi‐EGF‐IT in overcoming resistance to current EGFR inhibitors, and potential synergistic effects with immune checkpoint inhibitors. EGFR overexpression is also common in other cancers, such as lung and colon [43, 44]. Our report opens the possibility of testing bi‐EGF‐IT against other EGFR‐overexpressing cancers (e.g., lung cancer). We will also embark on investigational new drug (IND)‐enabling studies, including good manufacturing practice production and good laboratory practice preclinical toxicology studies.

## 5 Conclusions

In this study, we have developed a truncated DT‐based bi‐EGF‐IT with significantly improved efficacy and markedly less *in vivo* off‐target toxicity compared with its monovalent counterpart, mono‐EGF‐IT and erlotinib. Thus, the bi‐EGF‐IT is a promising novel drug candidate for further development in treating EGFR‐positive HNSCC.

## Conflict of interest

The authors declare no conflict of interest.

## Author contributions

ZQ and YQ as co‐first authors primarily performed the experiments and data analysis, and participated in writing the manuscript; ZW (Zhaohui), HZ, LL, and YL participated in the experiments; DG participated in the statistical analysis of the data; DM and EAP participated in the data analysis and writing the manuscript; and ZW (Zhirui) and SL as co‐corresponding authors primarily designed the project, analyzed the data, and wrote the manuscript.

### Peer Review

The peer review history for this article is available at https://publons.com/publon/10.1002/1878‐0261.12919.

## Supporting information


**Fig. S1.** *K*_D_ determination for mono‐EGF‐IT and bi‐EGF‐IT in 13 human EGFR^+^ HNSCC cell lines.
**Fig. S2.** *K*_D_ comparison between mono‐EGF‐IT and bi‐EGF‐IT in 14 EGFR^+^ HNSCC cell lines.
**Fig. S3.** *In vitro* efficacy of human EGF fusion toxins against 13 human EGFR^+^ HNSCC cell lines determined by the CellTiter‐Glo® Luminescent Cell Viability Assay.
**Fig. S4.** *In vitro* efficacy of erlotinib in 14 human EGFR^+^ HNSCC cell lines using the CellTiter‐Glo® Luminescent Cell Viability Assay.
**Fig. S5.** Necropsy results for *NSG* mice treated with mono‐EGF‐IT or bi‐EGF‐IT.
**Table S1.** HNSCC cell lines used in this study.
**Table S2.** Antibodies used in this study.
**Table S3.** PCR primers used in this study.
