# Supplementary material for: A novel diphtheria toxin‐based bivalent human EGF fusion toxin for treatment of head and neck squamous cell carcinoma
Source: Mol Oncol. 2021 Feb 20;15(4):1054–68. doi: 10.1002/1878-0261.12919 (PMC8024719; doi:10.1002/1878-0261.12919)
Supplement: Supplementary file 1 — Fig. S1. K D determination for mono‐EGF‐IT and bi‐EGF‐IT in 13 human EGFR+ HNSCC cell lines. Fig. S2. K D comparison between mono‐EGF‐IT and bi‐EGF‐IT in 14 EGFR+ HNSCC cell lines. Fig. S3. In vitro efficacy of human EGF fusion toxins against 13 human EGFR+ HNSCC cell lines determined by the CellTiter‐Glo® Luminescent Cell Viability Assay. Fig. S4. In vitro efficacy of erlotinib in 14 human EGFR+ HNSCC cell lines using the CellTiter‐Glo® Luminescent Cell Viability Assay. Fig. S5. Necropsy results for NSG mice treated with mono‐EGF‐IT or bi‐EGF‐IT. Table S1. HNSCC cell lines used in this study. Table S2. Antibodies used in this study. Table S3. PCR primers used in this study. [file MOL2-15-1054-s001.pdf]

Fig. S1

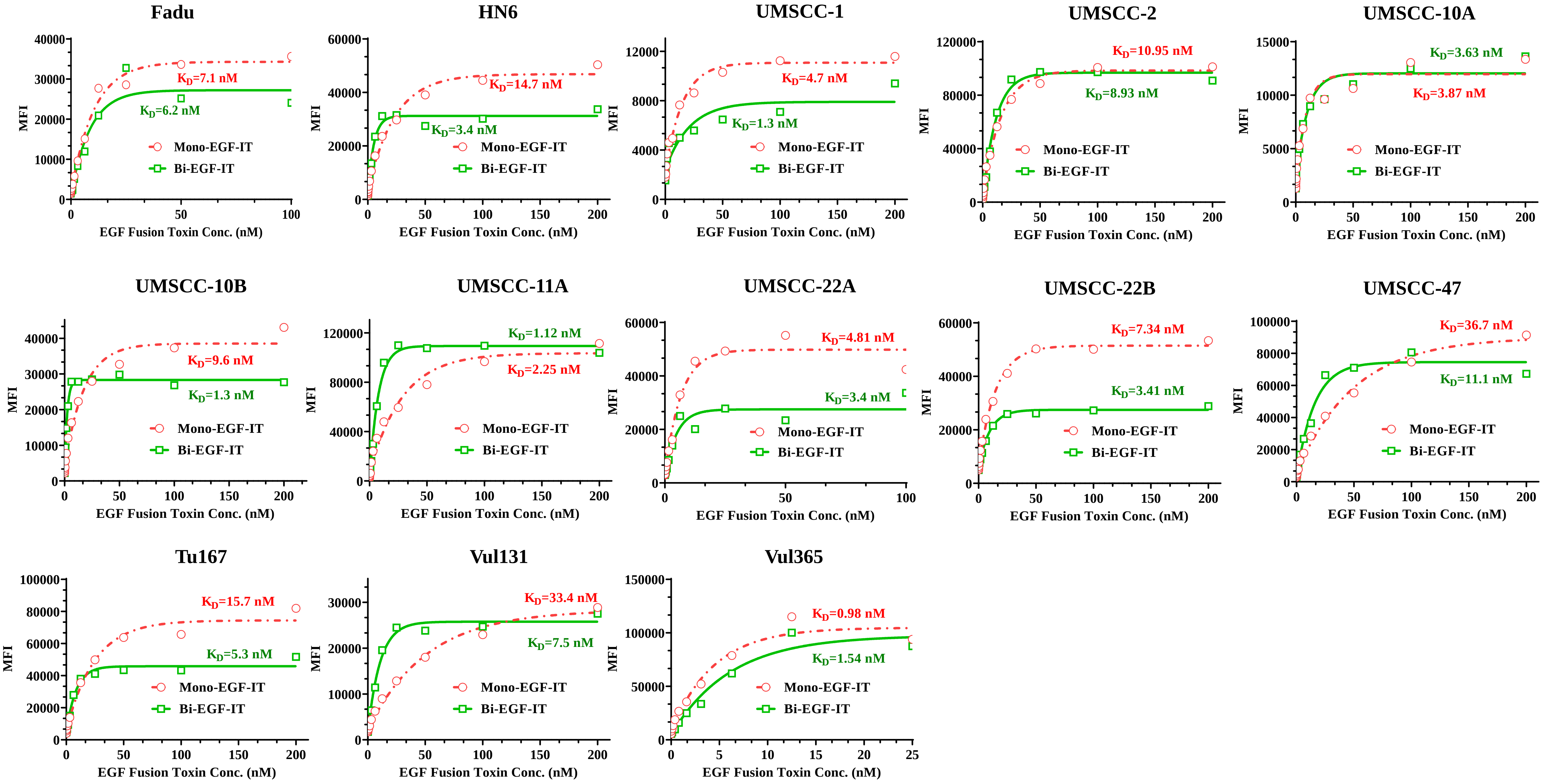

Fadu
KD=7.1 nM
KD=6.2 nM
HN6
KD=14.7 nM
KD=3.4 nM
UMSCC-1
KD=4.7 nM
KD=1.3 nM
UMSCC-2
KD=10.95 nM
KD=8.93 nM
UMSCC-10A
KD=3.63 nM
KD=3.87 nM
UMSCC-10B
KD=9.6 nM
KD=1.3 nM
UMSCC-11A
KD=1.12 nM
KD=2.25 nM
UMSCC-22A
KD=4.81 nM
KD=3.4 nM
UMSCC-22B
KD=7.34 nM
KD=3.41 nM
UMSCC-47
KD=36.7 nM
KD=11.1 nM
Tu167
KD=15.7 nM
KD=5.3 nM
Vul131
KD=33.4 nM
KD=7.5 nM
Vul365
KD=0.98 nM
KD=1.54 nM
Mono-EGF-IT
Bi-EGF-IT
MFI
EGF Fusion Toxin Conc. (nM)

Fig. S1. K_D determination for mono-EGF-IT and bi-EGF-IT in 13 human $EGFR^+$ HNSCC cell lines. Anti-human EGFR mAb was included as a positive control, and biotinylated anti-murine PD-1 immunotoxin was used as a negative background control for protein biotinylation. The data are representative of three individual experiments. K_D was determined using flow cytometry and nonlinear least-squares fitting. The mean fluorescence intensity (MFI) was plotted over a wide range of biotinylated mono-EGF-IT or bi-EGF-IT concentrations. Nonlinear regression was based on the equation $Y = Bmax * X/(K_D + X)$, where Y = MFI at the given biotinylated fusion toxin concentration after subtracting the background, X = biotinylated fusion toxin concentration, and Bmax = the maximum specific binding in the same units as Y.

Fig. S2

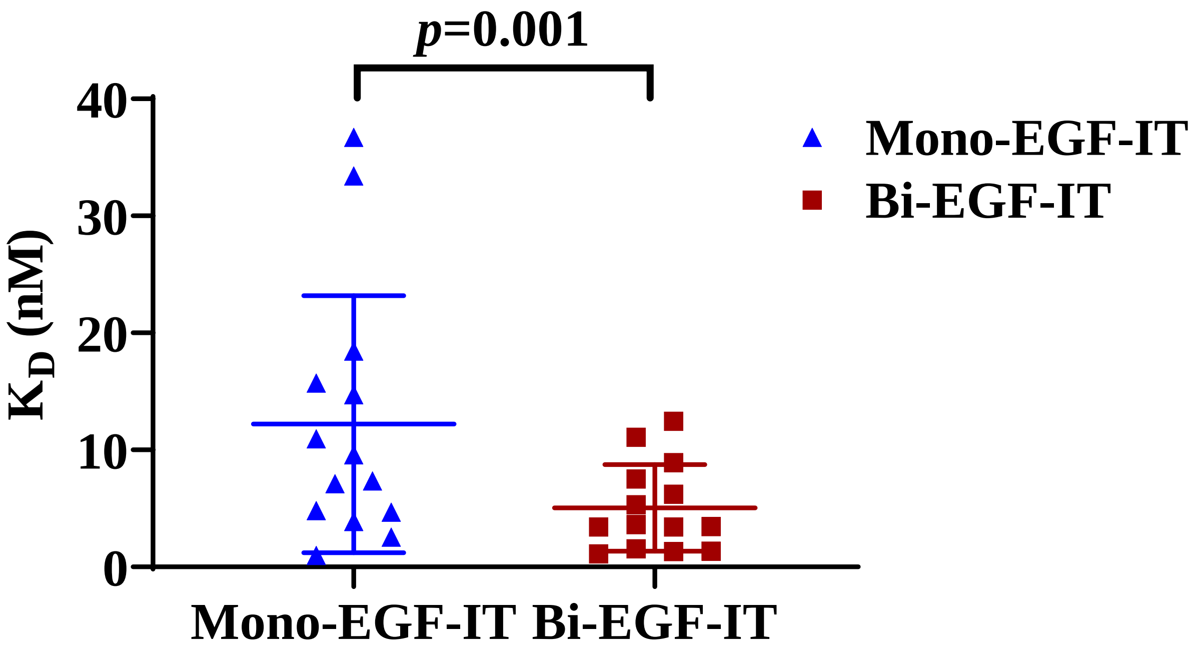

p=0.001
40
30
20
10
0
K_D (nM)
Mono-EGF-IT
Bi-EGF-IT
Mono-EGF-IT
Bi-EGF-IT

Fig. S2. K_D comparison between mono-EGF-IT and bi-EGF-IT in 14 $EGFR^+$ HNSCC cell lines. Because the distribution of the K_D values from all the cell lines was skewed, the non-parametric Wilcoxon signed-rank test was carried out to test the null hypothesis of no difference between the K_D values from the mono-EGF-IT and bi-EGF-IT groups.

Fig. S3

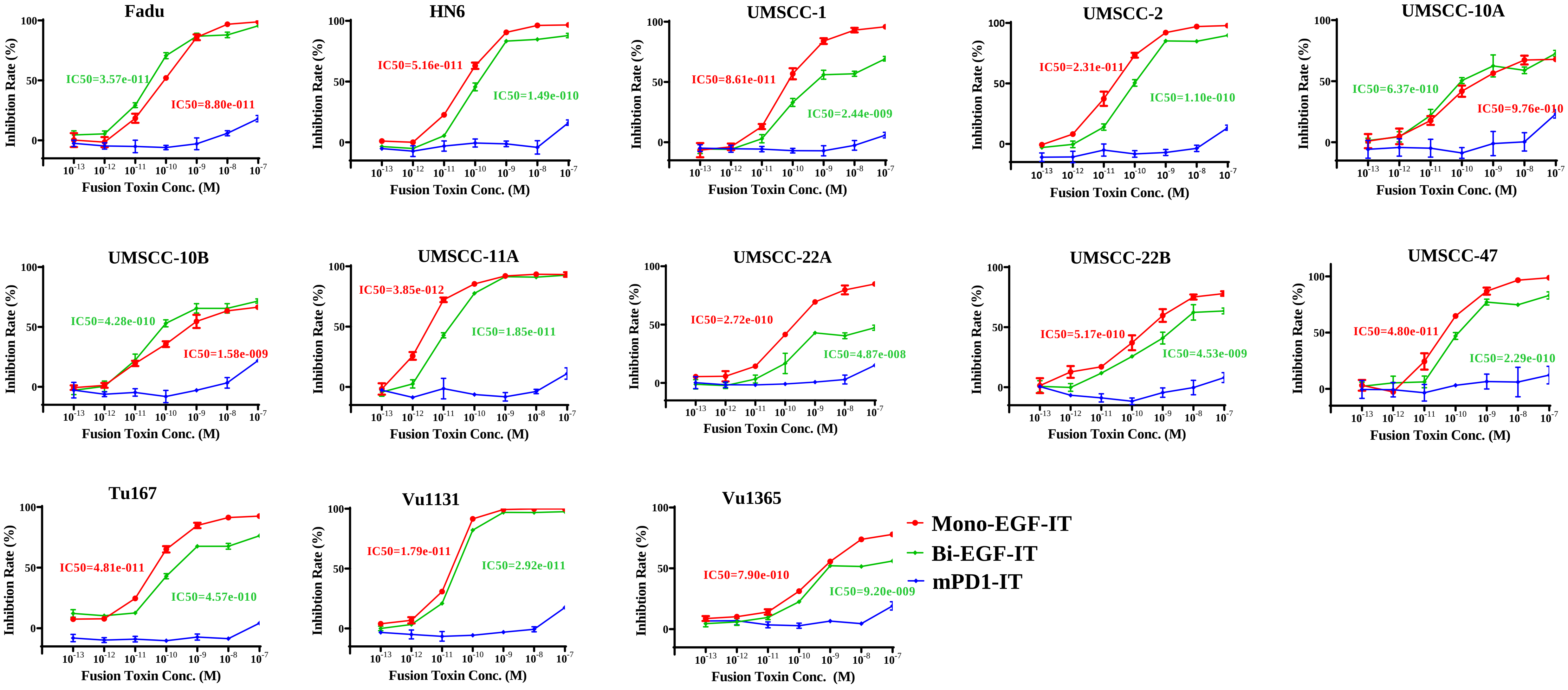
Fadu
IC50=3.57e-011
IC50=8.80e-011
HN6
IC50=5.16e-011
IC50=1.49e-010
UMSCC-1
IC50=8.61e-011
IC50=2.44e-009
UMSCC-2
IC50=2.31e-011
IC50=1.10e-010
UMSCC-10A
IC50=6.37e-010
IC50=9.76e-010
UMSCC-10B
IC50=4.28e-010
IC50=1.58e-009
UMSCC-11A
IC50=3.85e-012
IC50=1.85e-011
UMSCC-22A
IC50=2.72e-010
IC50=4.87e-008
UMSCC-22B
IC50=5.17e-010
IC50=4.53e-009
UMSCC-47
IC50=4.80e-011
IC50=2.29e-010
Tu167
IC50=4.81e-011
IC50=4.57e-010
Vu1131
IC50=1.79e-011
IC50=2.92e-011
Vu1365
IC50=7.90e-010
IC50=9.20e-009
Inhibtion Rate (%)
Fusion Toxin Conc. (M)
Mono-EGF-IT
Bi-EGF-IT
mPD1-IT

Fig. S3. *In vitro* efficacy of human EGF fusion toxins against 13 human $EGFR^+$ HNSCC cell lines determined by the CellTiter-Glo® Luminescent Cell Viability Assay (red line: mono-EGF-IT group; green line: bi-EGF-IT group; blue line: Anti-murine PD-1 immunotoxin group as the negative control). Y-axis: percent inhibition of the cell viability by determining the number of viable cells based on the quantification of ATP. X-axis: fusion toxin concentration. Cycloheximide (1.25 mg/mL) was used as a positive control. The negative control wells contained cells without fusion toxin. Data are from three individual experiments for each HNSCC cell line. Error bars indicate SD.

Fig. S4

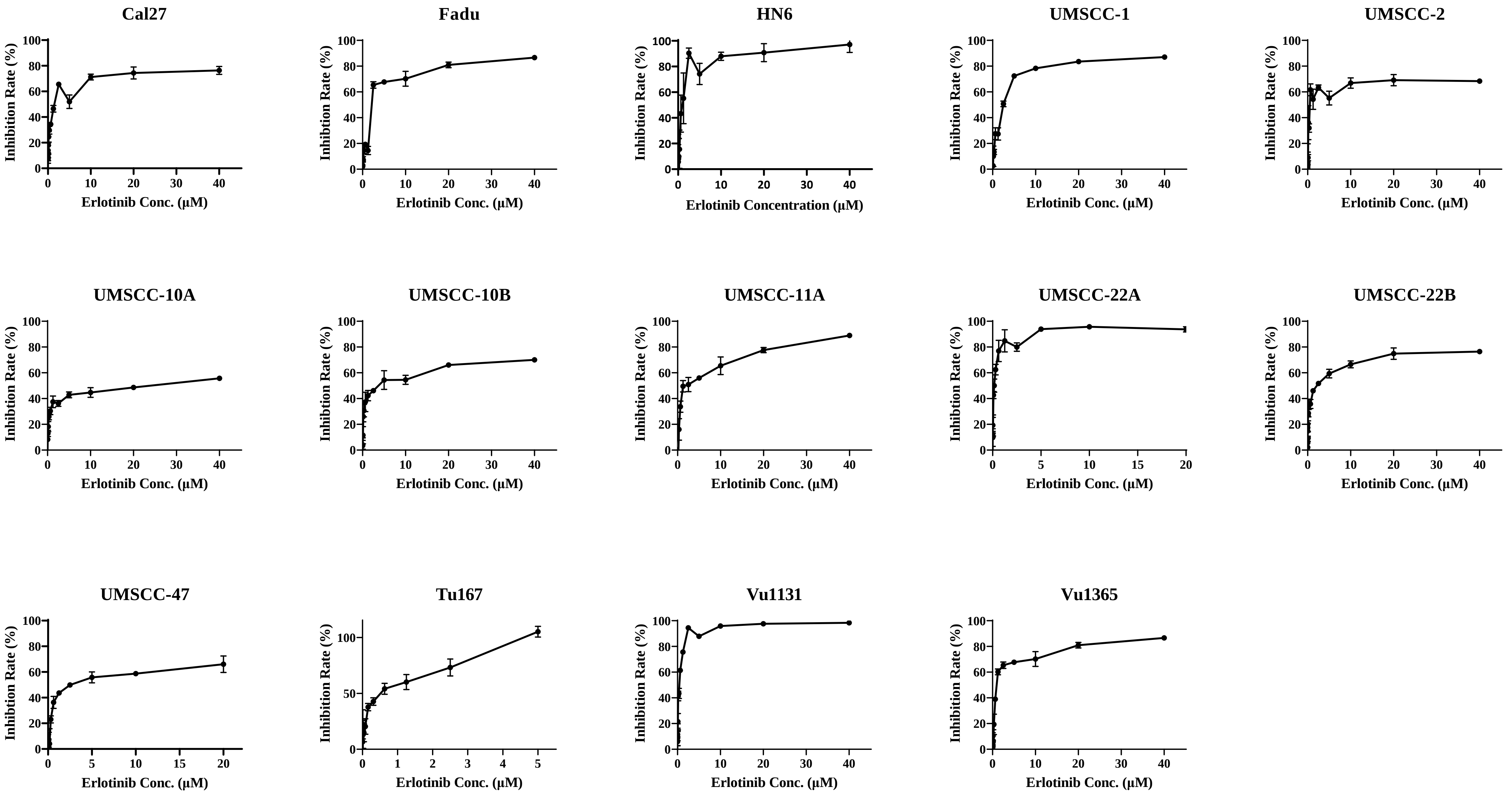
Cal27
Fadu
HN6
UMSCC-1
UMSCC-2
UMSCC-10A
UMSCC-10B
UMSCC-11A
UMSCC-22A
UMSCC-22B
UMSCC-47
Tu167
Vu1131
Vu1365
Inhibition Rate (%)
Erlotinib Conc. (μM)
Erlotinib Concentration (μM)

Fig. S4. *In vitro* efficacy of erlotinib in 14 human $EGFR^+$ HNSCC cell lines using the CellTiter-Glo® Luminescent Cell Viability Assay. Y-axis: percent inhibition of cell viability determined by the number of viable cells based on the quantification of the ATP present. X-axis: plated erlotinib concentration. Cycloheximide (1.25 mg/mL) was used as a positive control. The negative control contained cells without erlotinib. Data were from three individual experiments for each HNSCC cell line. Error bars indicate SD.

Fig. S5

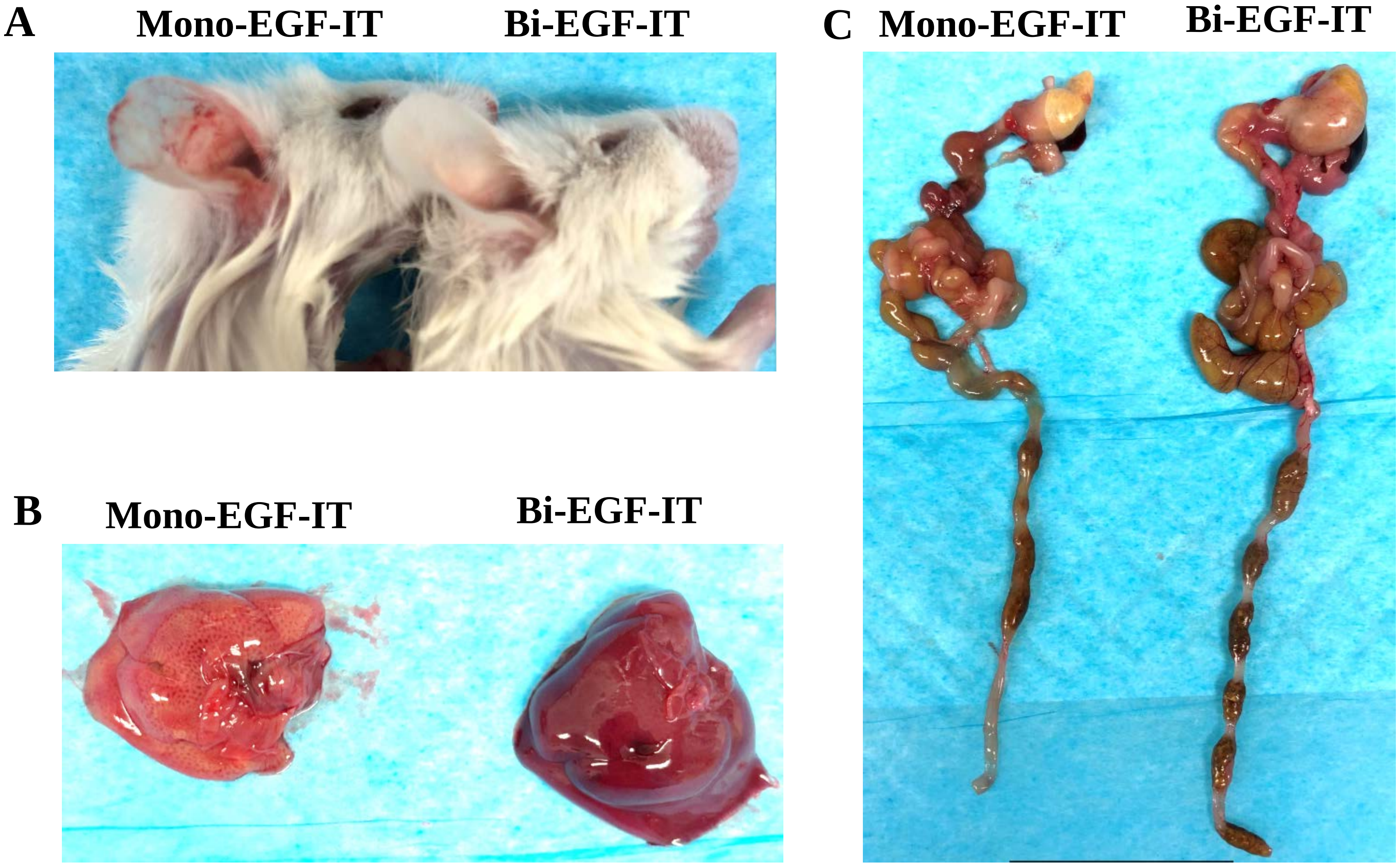
A
Mono-EGF-IT
Bi-EGF-IT
B
Mono-EGF-IT
Bi-EGF-IT
C
Mono-EGF-IT
Bi-EGF-IT

Fig. S5. Necropsy results for *NSG* mice treated with mono-EGF-IT or bi-EGF-IT. *NSG* mice were treated with mono-EGF-IT or bi-EGF-IT without tumor cell injection. Mice were euthanized on day 13 after the first injection of the fusion toxins. Gross changes for the ear (A), liver (B), and gastrointestinal tract (C) are shown.

Table S1. HNSCC cell lines used in this study

Cell Lines	Location	Gender	Age	HPV	p53 mutation	Fanconi Anemia	TNM	Stage	Resources
Cal27	Tongue	Male	56		His193Leu				ATCC
FaDu	Hypopharynx	Male	56		Arg248Leu				ATCC
HN6	Tongue	Male	54				T2N0M0		ATCC
UMSCC-1	floor of mouth	Male	72	negative	no		T2NoMo	II	UM
UMSCC-2	alveolar ridge	Female	64				T2NoMo	II	UM
UMSCC-10A	larynx (true vocal cord)	Male	57		Gly245Cys		T3N0M0	III	UM
UMSCC-10B	larynx-neck lymph node	Male	58		Gly245Cys		T3N1M0	III	UM
UMSCC-11A	Larynx	Male	64		no				UM
UMSCC-11B	larynx-neck lymph node	Male	64						UM
UMSCC-22A	Hypopharynx	female	59	negative	Tyr220Cys		T2N1M0	III	UM
UMSCC-22B	hypopharynx-neck lymph node	female	59	negative	Tyr220Cys		T2N1M0	III	UM
UMSCC-47	tongue	Male	53	positive	no				UM
Tu167	floor of mouth	Male	72						ATCC
Vu1131	floor of mouth	Female	34	negative	R273L	FA-C, c.67delG	T4N2b		Vrije University
Vu1365	Mouth	Male	22	negative	R282W	FA-A			Vrije University

FA: Fanconi Anemia, UM: University of Michigan.

Table S2. Antibodies used in this study

Antibody Name	Clone#	Source	Cat#
PE-mouse anti-human EGFR	EGFR.1	BD	555997
PE-streptavidin	L200	Biolegend	405204
PE Mouse IgG2b, κ Isotype Ctrl Antibody	MPC-11	Biolegend	400313
7-Aminoactinomycin (7-AAD)	SP-34-2	Sigma	A9400
His Tag Antibody (Mouse)	6G2A9	GenScript	A00186
Diphtheria Toxin Antibody (Mouse)		Meridian Life Science	C86036M
Rat anti-Mouse IgG (H+L) Secondary Antibody, HRP	LO-MG-7	Invitrogen	04-6020

Table S3. PCR primers used in this study

---

hEGF-Nco
5' C ATG <u>CCA TGG</u> GGT GGT GGT GGT TCT AAC TCT GAC TCC GAG TGT CCA 3'
*Nco*I

hEGF-Bam1
5' CGG <u>GGA TCC</u> ACC ACC ACC AGA ACC ACC ACC ACC TCT CAA CTC CCA CCA CTT CAA 3'
*Bam*HI

hEGF-Bam2
5' CGG <u>GGA TCC</u> GGT GGT GGT GGT TCT AAC TCT GAC TCC GAG TGT CCA 3'
*Bam*HI

hEGF-Eco
5' CCG <u>GAA TTC</u> TTA GTG GTG GTG GTG GTG GTG TCT CAA CTC CCA CCA CTT CAA 3'
*Eco*RI
